# Mutation spectrum of *RB1* mutations in retinoblastoma cases from Singapore with implications for genetic management and counselling

**DOI:** 10.1371/journal.pone.0178776

**Published:** 2017-06-02

**Authors:** Swati Tomar, Raman Sethi, Gangadhara Sundar, Thuan Chong Quah, Boon Long Quah, Poh San Lai

**Affiliations:** 1 Department of Paediatrics, Yong Loo Lin School of Medicine, National University of Singapore, Singapore, Singapore; 2 Department of Ophthalmology, National University Hospital, Singapore, Singapore; 3 Singapore National Eye Centre, Singapore, Singapore; NIDCR/NIH, UNITED STATES

## Abstract

Retinoblastoma (RB) is a rare childhood malignant disorder caused by the biallelic inactivation of *RB1* gene. Early diagnosis and identification of carriers of heritable *RB1* mutations can improve disease outcome and management. In this study, mutational analysis was conducted on fifty-nine matched tumor and peripheral blood samples from 18 bilateral and 41 unilateral unrelated RB cases by a combinatorial approach of Multiplex Ligation-dependent Probe Amplification (MLPA) assay, deletion screening, direct sequencing, copy number gene dosage analysis and methylation assays. Screening of both blood and tumor samples yielded a mutation detection rate of 94.9% (56/59) while only 42.4% (25/59) of mutations were detected if blood samples alone were analyzed. Biallelic mutations were observed in 43/59 (72.9%) of tumors screened. There were 3 cases (5.1%) in which no mutations could be detected and germline mutations were detected in 19.5% (8/41) of unilateral cases. A total of 61 point mutations were identified, of which 10 were novel. There was a high incidence of previously reported recurrent mutations, occurring at 38.98% (23/59) of all cases. Of interest were three cases of mosaic *RB1* mutations detected in the blood from patients with unilateral retinoblastoma. Additionally, two germline mutations previously reported to be associated with low-penetrance phenotypes: missense-c.1981C>T and splice variant-c.607+1G>T, were observed in a bilateral and a unilateral proband, respectively. These findings have implications for genetic counselling and risk prediction for the affected families. This is the first published report on the spectrum of mutations in RB patients from Singapore and shows that further improved mutation screening strategies are required in order to provide a definitive molecular diagnosis for every case of RB. Our findings also underscore the importance of genetic testing in supporting individualized disease management plans for patients and asymptomatic family members carrying low-penetrance, germline mosaicism or heritable unilateral mutational phenotypes.

## Introduction

Retinoblastoma (RB) is a retinal cancer associated with biallelic loss of *RB1* gene. The global incidence of this disease is 1 case in 15,000 to 20,000 live births [1] with the average annual incidence in Singapore reported as 2.4 to 11.1 cases per million children [2,3] occurring equally among males and females [4]. On a global scale, an estimated 3001 to 3376 children die due to retinoblastoma annually [1]. The mortality rate in Asia (39%) is much higher than that of Europe, Canada, and the USA (3–5%) [1] due to the gap in healthcare access which primarily refers to the fact that majority of RB patients are diagnosed in low‑ and middle‑income countries, whereas the bulk of retinoblastoma-specific health care facilities are available in high‑income countries [5]. In more developed countries in Asia such as in Singapore, the overall 5-year survival rate can be much higher ranging between 88.1% to 91% [3,4].

Most RB cases are diagnosed by 5 years of age and occur in either heritable or non-heritable forms. Non-heritable RB arises from somatic mutations occurring on both alleles of *RB1* gene in the developing retina, whereas heritable RB arises from the inheritance of at least one germline mutation along with an acquired *RB1* somatic mutation [6]. All bilateral retinoblastomas are heritable, of which about 10% are inherited [6,7]. Fifteen percent of unilateral retinoblastoma occur due to *de novo* germline *RB1* mutations which is transmissible in subsequent generations [8]. In heritable RB, offspring have a 50% chance of inheriting the mutant *RB1* allele from an affected parent. Such an inheritance of the mutant *RB1* allele results in a 97% risk of developing the disease and a high lifelong risk of secondary cancers [8].

*RB1* inactivation has been implicated in more than 97% of all RB cases with mutations in this gene being undetectable in the remaining cases [5]. Recent reports suggest that other genes may play a role in either driving tumor initiation or progression [9,10]. It has been postulated that probable candidate genes may be located in chromosomal regions with recurrent gains [11–15] and losses [16–18] observed in RB tumors. Rushlow et al provided evidence that retinoblastoma could also be caused by *MYCN* oncogene amplification and predicted that 18% of cases who are diagnosed with non-familial unilateral RB before the age of 6 months would harbour only *MYCN* amplification and no *RB1* mutations [9]. They also quoted another 1.5% of unilateral non-familial RB whose pathogenesis could not be explained as they harboured normal *RB1* and *MYCN* genes.

Genetic testing in RB is essential to not only identify the spectrum of underlying mutations but also to delineate heritable RB for non-heritable ones for efficient genetic counselling [5]. Hence, this study aims to characterize the spectrum of *RB1* mutations in RB cases seen among patients in Singapore in order to aid disease management.

## Materials and methods

### Patients

This study was conducted on DNA samples from a cohort of 59 retinoblastoma cases (18 bilateral and 41 unilateral), collected over a period of 15 years. Diagnosis of retinoblastoma was established by standard ophthalmologic and histological criteria. Thirty-four cases were female and twenty-five were male. When an *RB1* mutation was found in the peripheral blood of the proband, DNA samples from the parents were tested for presence of the identified mutation. If parents tested positive for the proband’s mutation, siblings’ blood were collected and analysed similarly. In addition, parental DNA was sought in cases where a gross deletion in *RB1* gene was identified, to determine the parental origin of the loss of *RB1* allele. Samples from all patients and family members were collected with written informed consent and in accordance with the principles of the Declaration of Helsinki. The study was approved by the institutional review board of the National University of Singapore.

### DNA isolation

DNA samples used were extracted from matched peripheral blood (10ml in EDTA tubes) and fresh tumor samples (100–200 mg), collected after enucleation. DNA isolation protocol was adapted from the high salt extraction method of Miller et al [19].

### *RB1* gene sequencing

The DNA obtained from all 59 tumors and corresponding blood samples was sequenced for 27 exons and promoter region of *RB1* gene after PCR amplification using 27 sets of primers as described previously [20]. Some cases were sent out to an international laboratory (Impact Genetics Inc., Canada) for *RB1* gene sequence analysis and Allele-specific PCR (AS-PCR) for eleven recurrent *RB1* mutations. Additional information about *RB1* gene mutations were confirmed from gene locus specific mutation database (rb1-lsdb) and The Human Gene Mutation Database (HGMD). Predictive analysis tools were used to determine the pathogenicity status of novel variants. Missense mutations were analyzed by SIFT (http://sift.jcvi.org/www/SIFT_BLink_submit.html), CADD (http://cadd.gs.washington.edu/score) and Mutation taster (http://www.mutationtaster.org/), while all frameshift variants were predicted by PROVEAN (http://provean.jcvi.org) and Mutation Taster, respectively.

### Gross *RB1* deletions analysis

#### Multiplex ligation-dependent probe amplification (MLPA) analysis

To screen for deletions or duplications in the *RB1* gene, MLPA analysis was done using the SALSA MLPA kit P047-B1 *RB1* (MRC-Holland, Amsterdam, the Netherlands) according to the manufacturer’s protocol with 100 ng of genomic DNA from matched tumor and blood. The PCR amplicons were seperated on Genetic Analyzer 3130 (Applied Biosystems, Foster City, CA), and the results were analyzed using Cofflalyser Software available at http://www.mlpa.com/coffalyser/. Based on the normalized signal value ratio of 1:1; threshold ratios of 0.75 (deletion) and 1.30 (duplication) were used to indicate loss or gain of probe copy numbers respectively.

#### Microsatellite analysis and SNP genotyping

The extent of loss of heterozygosity (LOH) was assayed in matched tumor and blood DNA using 20 flanking extragenic microsatellite markers (S1 Table). Allelic imbalance affecting *RB1* gene locus at 13q14 was examined using three intragenic microsatellites: D13S153—located within intron 2 of *RB1*, dinucleotide repeats (TG)_22_—located within intron 4 and tetra nucleotide repeats (TTCT)_16_—located within intron 20 of *RB1* along with four previously reported SNP markers [21–24] (S2 Table). The SNP markers and Microsatellite markers were typed using standard PCR-based methods as described previously [17] and samples were scored as informative if the lymphocyte DNA showed heterozygosity of alleles for each marker, or non-informative for homozygosity or positive for LOH when the tumour showed complete loss of one allele [25]. LOH was ascertained when loss of one of the alleles in the tumour samples was observed whereas the matched lymphocyte sample showed heterozygous alleles.

### Methylation specific PCR (MSP)

Methylation analysis at the CpG islands of *RB1* Promoter in tumor and blood was analyzed using CpGenomeTM DNA Modification kit (Intergen) and methylation specific PCR using specific primers as previously described [26–28]. For *MGMT* promoter hypermethylation analysis, primers were synthesized using Primo MSP 3.4 software (http://www.changbioscience.com/primo/primom.html) based on *MGMT* promoter sequence (GenBank Acc. No. X61657). MSP was performed in two separate reactions to identify unmethylated and methylated DNA as described previously [28].

### Quantitative multiplex PCR (QM-PCR)

QM-PCR studies were performed on tumor samples to determine copy number of *TNF* (6p21.3) using methods previously described [29–31]. A positive control, DNA from the WERI-*RB1* retinoblastoma cell line which has isochromosome 6p (i6p) and therefore carrying 4 copies of the chromosomal region 6p [32], and a normal DNA as external control was amplified together with tumour samples in each PCR reaction. The PCR products were prepared as described previously for genotyping on the ABI PRISM 3130xl Sequencer[17]. The results were analyzed using the GeneMapper^®^ Software v4.0. The copy number in tumour sample was compared with the normal and positive control sample in each case. The range for diploid or normal two-copy number was calculated using a series of normal DNA samples. The values obtained from QM-PCR of the WERI-*RB1* cell line were used to indicate minimum value above which more than two copies of the gene are expected in the test sample.

### Statistics

To determine differences in the frequencies of observed types of *RB1* point mutations in our cohort and those from worldwide mutation frequencies from a reported meta-analysis by Valverde et al 2005 [33], the χ2 goodness-of-fit test was performed. Fisher’s exact t test was performed to test the significance of all contingency tables in the study. Welch’s t test was done to test the significance of age distribution by different categories. P value of <0.05 was considered significant.

## Results

### Age at diagnosis

**Fig 1:**
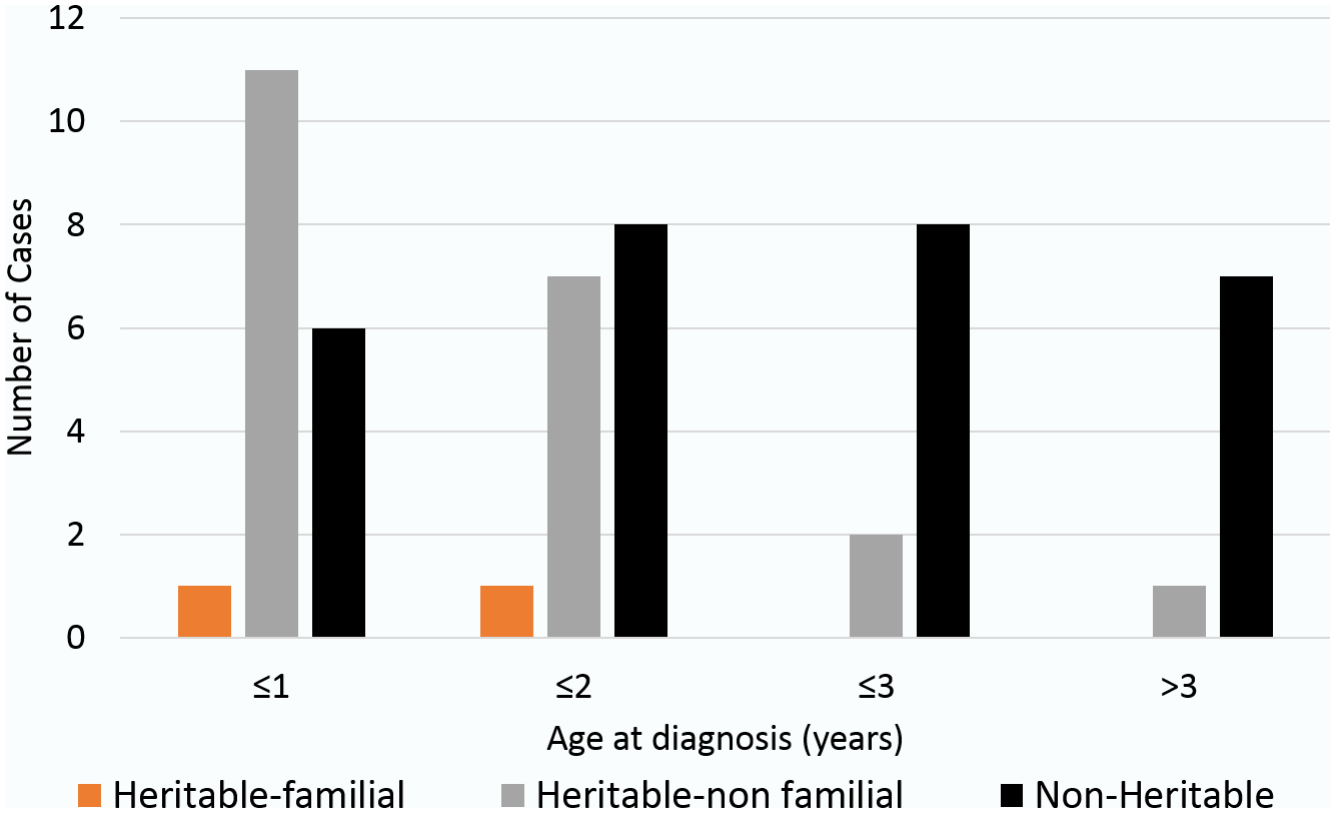
Age distribution of heritable and non-heritable RB cases. The age distribution between Heritable-familial, Heritable-non familial and Non-Heritable RB cases was significant by Welch’s t test of unpaired groups (p = 0.001351; 95% CI [-22.4729, -5.7665]). Three cases had unknown age at diagnosis and hence not reflected.

The mean age at presentation of the disease in our data set of 59 cases (22.1± 16.5 months) was slightly lower than what was reported in a previous clinical study on 51 Singaporean patients (25.7± 19.9 months) [4]. However, the overall frequency of bilateral cases in our study (30.5%, 18/59) was similar to the above study (31.4%, 16/51) [4]. A summary of cases by their respective clinicopathological characteristics is given in Table 1. When the age of patients with somatic point mutations (25 unilateral cases) was compared to those with germline point mutations (18 bilateral + 7 unilateral cases), the distribution was found to be statistically significant by Welch’s t test of unpaired groups (Somatic group: mean age at diagnosis = 28.71 months, standard deviation = 18.51 months; Germline group: mean age at diagnosis = 14.71, standard deviation = 11.46 months; p = 0.001824, 95% CI [-22.5448, -5.469]). The overall trend of age at diagnosis for the three categories of patients, as given in Fig 1, shows that the patients with heritable mutational events (Familial and Non-Familial) were diagnosed earlier than those with non-heritable mutations (Welch’s t test, p = 0.001351). We did not have age at diagnosis available for three cases and hence those cases were not considered for this comparison. Additionally, patients with germline nonsense mutations presented at a younger age as compared to those with somatic nonsense mutations (p = 0.07244). Lastly, there was a significant correlation between earlier age of diagnosis for bilateral cases with point mutations to that of unilateral cases with point mutations (p = 0.02649; 95% CI [-18.301, -1.1901]). This correlation did not hold significant when compared by gender of the cases (p = 0.7733; 95% CI [-7.8444, 10.4827]).

**Table 1:** Clinicopathological distribution of heritable and non-heritable retinoblastoma patients by laterality, age at diagnosis and family history.

	Heritable	Non-Heritable	Uncategorized^#^	Total
**All patients**	25	31	3	**59**
**Laterality**				
Bilateral	17	1	0	**18**
Unilateral	8	30	3	**41**
Age at Diagnosis (months)*				
≤ 12	12	6	2	**20**
≤ 24	8	9	0	**17**
< 36	2	4	0	**6**
≥36	1	11	1	**13**
**Family History**				
Familial	2	0	0	**2**
Non-Familial	23	31	3	**57**

* Three cases had unknown age at diagnosis and hence not reflected.

^#^ No *RB1* mutations could be detected in these cases.

### Mutations identified in *RB1*

Among the 118 *RB1* alleles examined from the 59 RB cases, 98 mutant alleles were identified (83.05%) (Table 2). The percentage of mutated alleles identified within the unilateral and bilateral cases are shown in Table 2. The types of *RB1* mutations carried by these alleles were point mutations, gross deletions and promoter methylation.

**Table 2:** Mutant *RB1* alleles identified in all 59 RB cases.

	Unilateral	Bilateral	Total
**No. of probands analyzed**	41	18	**59**
**No. of alleles examined**	82	36	**118**
**No. of mutant alleles identified**	67	31	**98**
**% of identified mutant allele**	81.70%	86.11%	**83.05%**

#### Spectrum of *RB1* point mutations

**Fig 2:**
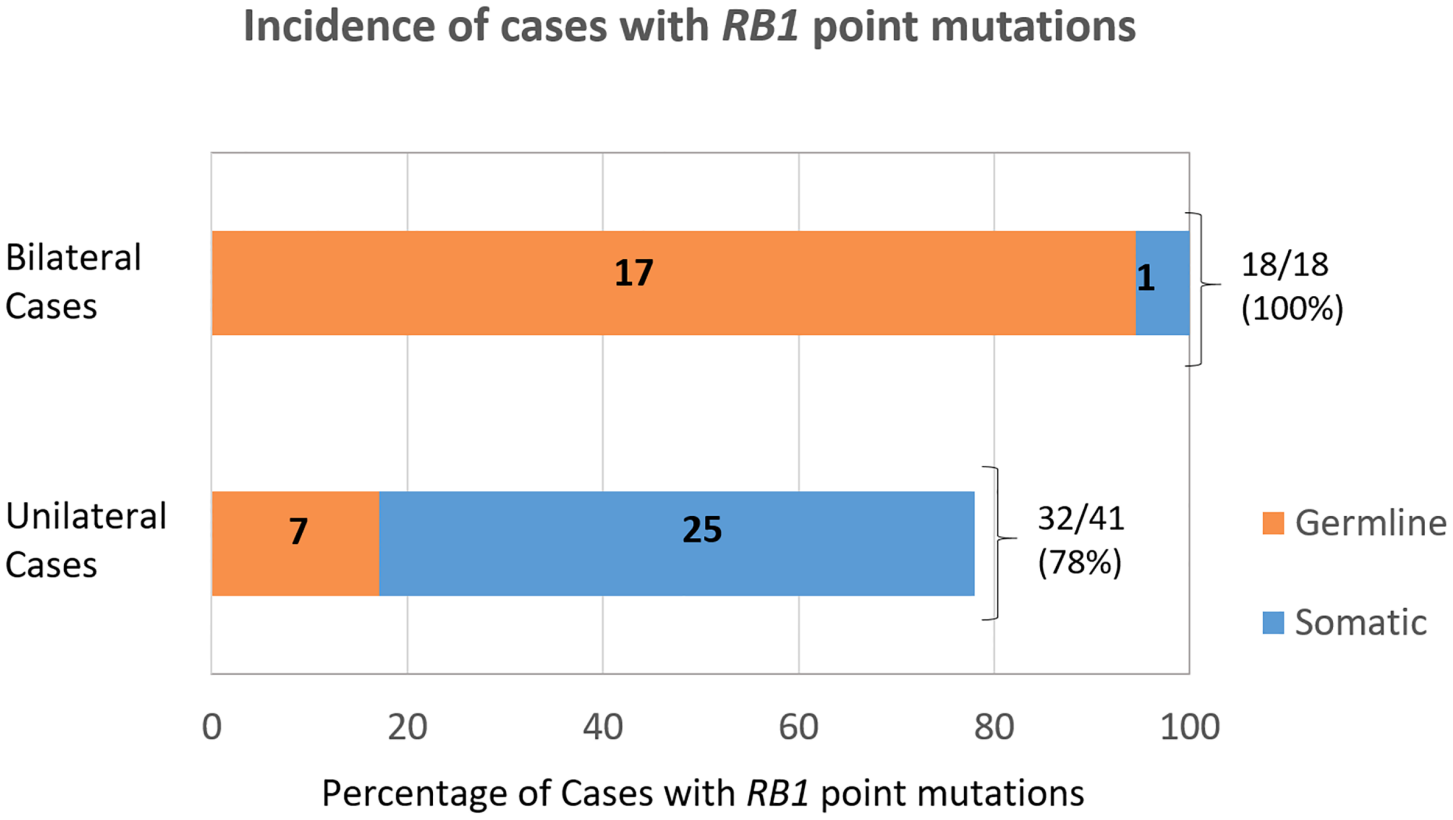
Incidence of germline and somatic *RB1* point mutations in 50 RB cases. A total of 61 *RB1* point mutations were identified in 50 RB probands. The distribution of mutations by type of tumor (unilateral and bilateral) and whether they were detected in blood (germline) or only tumor (somatic) is shown.

**Fig 3:**
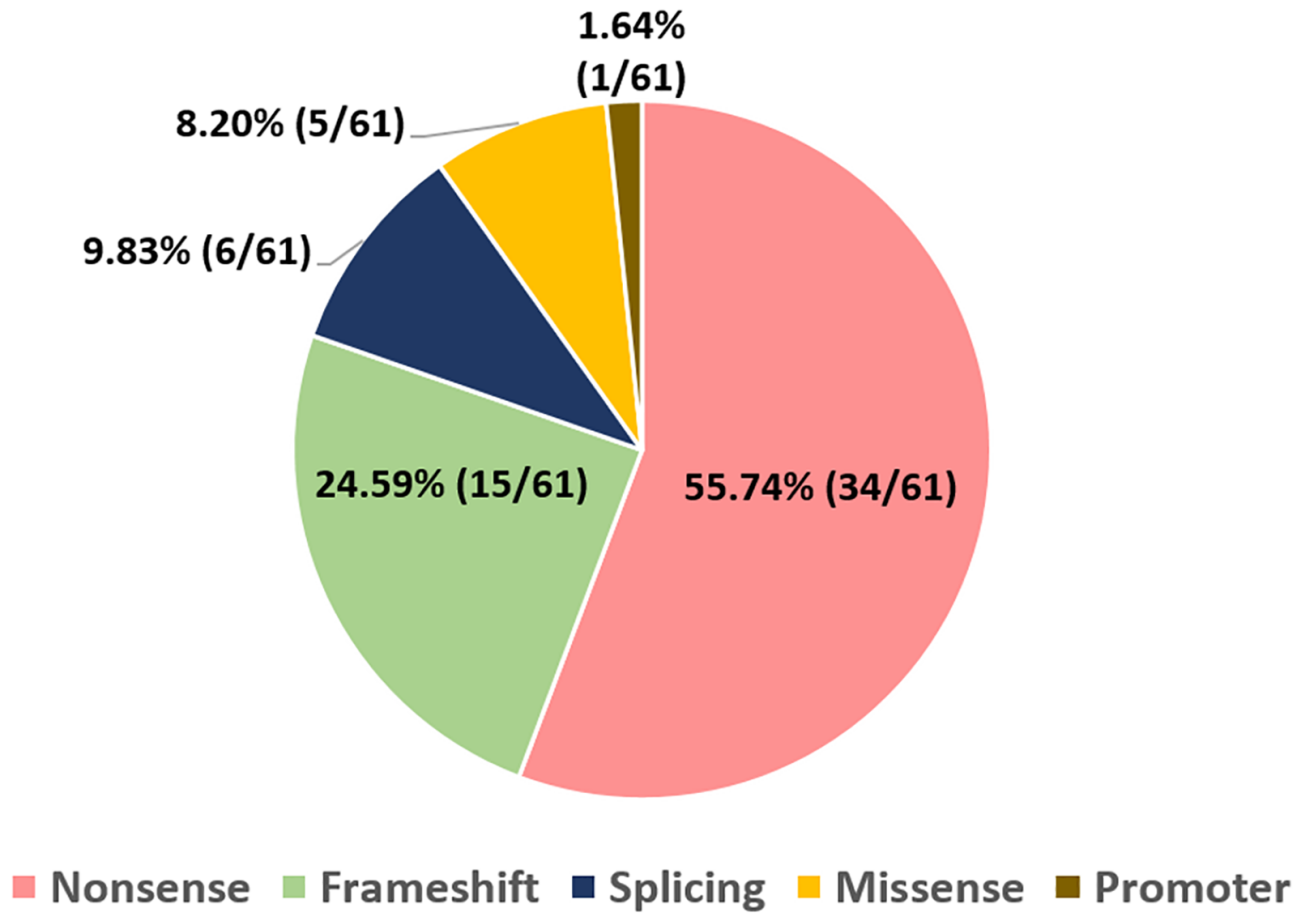
Distribution of total *RB1* point mutations by Type. The frequency of nonsense, frameshift, splice site, missense and promoter mutations among all the point mutations identified in our cohort.

**Fig 4:**
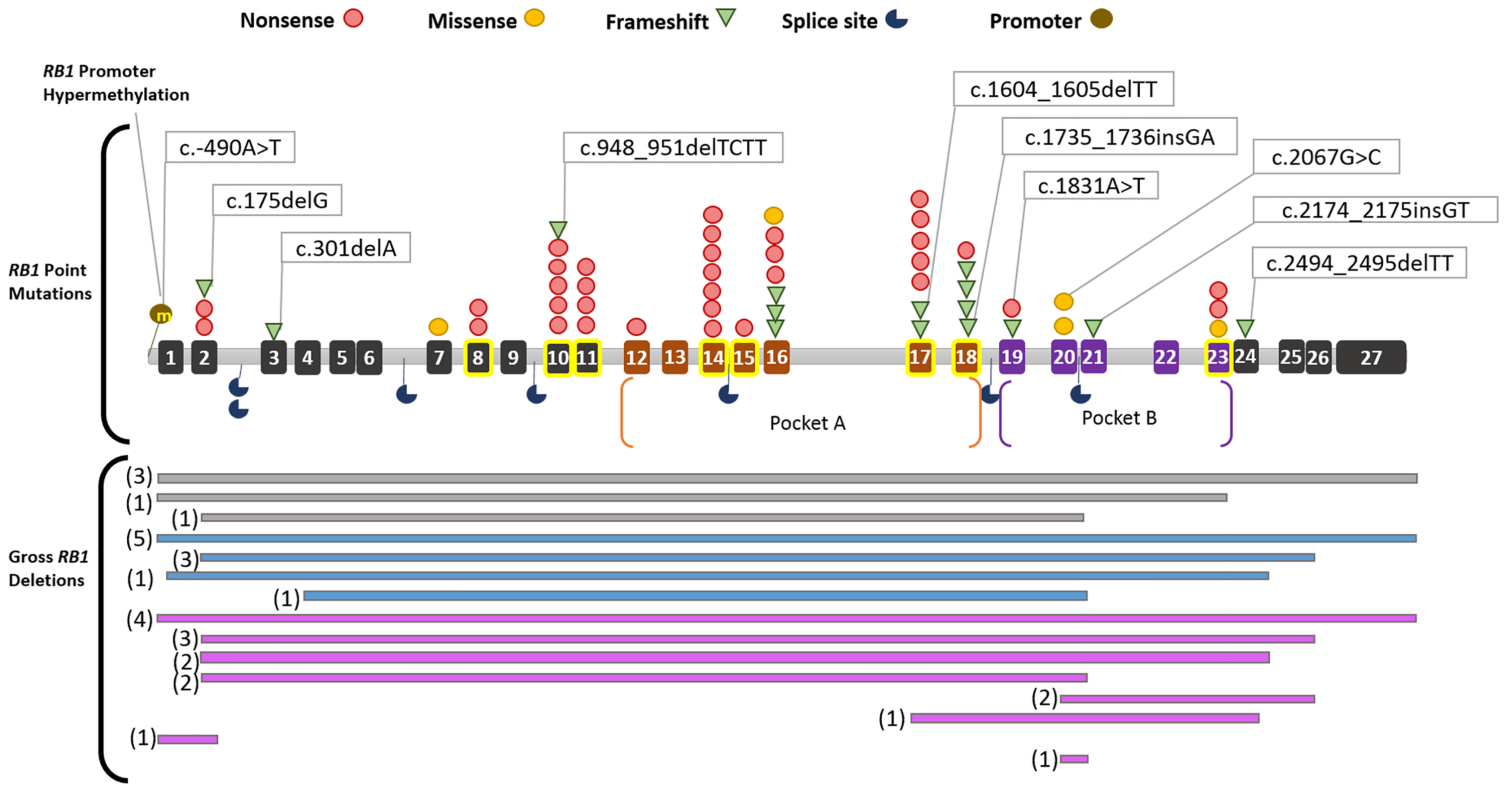
Schematic representation of sequence mutations across *RB1* gene. (GenBank Accession Number: L11910.1). The pocket domains are highlighted in orange (Pocket A) and purple (Pocket B) and exons are numbered respectively. Exons known to be mutational hotspots are highlighted with yellow boxes (Valverde et al 2005). Novel mutations are shown in callout boxes. Gross *RB1* deletions are shown in blue for paternal loss of allele and pink for maternal loss of allele. Grey indicates unknown inheritance. The respective frequencies of gross *RB1* deletions are given in brackets.

A total of 61 point mutations were identified in 84.7% (50/59) of all our retinoblastoma cases. This comprised 100% (18/18) of bilateral cases and 78% (32/41) of unilateral cases (Fig 2). The spectrum of different mutation types among the 61 point mutations were; nonsense mutations occurring at 55.7% (34/61), followed by 24.6% (15/61) frameshift, 9.8% (6/61) splicing, 8.2% (5/61) missense and 1.64% (1/61) promoter alterations (Fig 3). Exons coding for pocket domains of pRB, involved in regulation of transcription, (exons 12–18, domain A and 19–23, domain B) harbored 57.4% (35/61) of all point mutations. In our cohort, no mutations were observed for exons 1, 4, 5, 6, 9, 13, 22 and 25–27 (Fig 4). The graphical representation of all identified mutations spanning the entire *RB1* gene is shown in Fig 4. Ten novel *RB1* variants, representing 16.4% (10/61) of all the mutations identified were found in 3 bilateral (germline) and 7 unilateral (somatic) tumors (Table 3). All novel mutations except the promoter variant were predicted to be deleterious by four commonly used *in silico* analyses tools namely: PROVEAN, Mutation Taster, SIFT and CADD. The chi square test for independence for novel variants v/s known variants; germline v/s somatic variants and variants present in bilateral v/s unilateral cases did not show any statistical significance. In addition, 9 point mutations occurred more than once in 23 unrelated RB cases (16 unilateral and 7 bilateral) as shown in Table 4. These recurrent mutations comprised 44.3% (27/61) of all the identified point mutations. The most frequent recurring mutation was—p.Arg320* (Exon 10), which was found in five different unrelated cases. It was followed by p.Arg358* (Exon 11) and p.Arg455* (Exon 14) variants which occurred four times, respectively (Table 4). Another two mutations occurred three times, namely p.Arg445* (Exon 14) and p.Tyr498* (Exon 16). Lastly, four mutations: p.Arg579Glnfs*29 (Exon 18), p.Arg787* (Exon 23), p.Arg255* (Exon 8) and p.Arg552* (Exon 17); affected two cases each. The complete list of *RB1* mutations is given in S3 Table.

**Table 3:** Novel *RB1* point mutations.

Case	Codon Change	Protein Change	Type	Location	Prediction#
367T	c.175delG	*p*.Ala59Hisfs*5	Frameshift deletion	Exon 2	Deleterious (Mutation Taster, PROVEAN)
381T	c.2494_2495delTT	*p*.Leu832Serfs*5 (C-terminus)	Frameshift deletion	Exon 24	Deleterious (Mutation Taster, PROVEAN)
208T	c.1735_1736insGA	*p*.Gly581Lysfs*31	Frameshift insertion	Exon 18	Deleterious (Mutation Taster, PROVEAN)
420T	c.1831A>T	*p*.Arg611*	Nonsense	Exon 19	Deleterious (Mutation Taster, CADD)
182T	c.301delA	*p*.Ile101Serfs*9	Frameshift deletion	Exon 3	Deleterious (Mutation Taster, PROVEAN)
210T	c.948_951delTCTT	*p*.Ser318Asnfs*13	Frameshift deletion	Exon 10	Deleterious (Mutation Taster, PROVEAN)
122T	c.1604_1605delTT	*p*.Phe535Tyrfs*19	Frameshift deletion	Exon 17	Deleterious (Mutation Taster, PROVEAN)
224T	c.2174_2175insGT	-	Frameshift insertion	Exon 21	Deleterious (Mutation Taster, PROVEAN)
410T	c.-490A>T	-	Promoter	Upstream	No Prediction
414T	c.2067G>C	*p*.*Gln689His*	Missense	Exon 20	Deleterious (Mutation taster, CADD)

^#^Pathogenicity prediction was done using Mutation Taster (http://www.mutationtaster.org/) and PROVEAN (http://provean.jcvi.org)

**Table 4:** Recurrent *RB1* point mutations.

Case	cDNA Change	Putative Consequence	Mutation Type	Exon	Previously Reported LOVD ID
280T	c.958C>T	p.Arg320*	Nonsense	10	*RB1*_00072
332T	c.958C>T	p.Arg320*	Nonsense	10	*RB1*_00072
423T	c.1363C>T	p.Arg455*	Nonsense	14	*RB1*_00096
572T	c.1363C>T	p.Arg455*	Nonsense	14	*RB1*_00096
545T	c.1494T>G	p.Tyr498*	Nonsense	16	*RB1*_00314
583T	c.1494T>G	p.Tyr498*	Nonsense	16	*RB1*_00314
212T	c.2359C>T	p.Arg787*	Nonsense	23	*RB1*_00005
212T	c.1072C>T	p.Arg358*	Nonsense	11	*RB1*_00008
583T	c.1736_1745del10	p.Arg579Glnfs*29	Frameshift deletion	18	*RB1*_00014
545T	c.1736_1745del11	p.Arg579Glnfs*29	Frameshift deletion	18	*RB1*_00014
182T	c.1654C>T	p.Arg552*	Nonsense	17	*RB1*_00121
227T	c.763C>T	p.Arg255*	Nonsense	8	*RB1*_00063
578T	c.958C>T	p.Arg320*	Nonsense	10	*RB1*_00072
232T	c.1072C>T	p.Arg358*	Nonsense	11	*RB1*_00008
244T	c.1072C>T	p.Arg358*	Nonsense	11	*RB1*_00008
345T	c.1072C>T	p.Arg358*	Nonsense	11	*RB1*_00008
244T	c.1333C>T	p.Arg445*	Nonsense	14	*RB1*_00003
320T	c.1333C>T	p.Arg445*	Nonsense	14	*RB1*_00003
394T	c.1333C>T	p.Arg445*	Nonsense	14	*RB1*_00003
150T	c.1363C>T	p.Arg455*	Nonsense	14	*RB1*_00096
435T	c.1363C>T	p.Arg455*	Nonsense	14	*RB1*_00096
329T	c.1494T>G	p.Tyr498*	Nonsense	16	*RB1*_00314
440T	c.1654C>T	p.Arg552*	Nonsense	17	*RB1*_00121
304T	c.2359C>T	p.Arg787*	Nonsense	23	*RB1*_00005
575T	c.763C>T	p.Arg255*	Nonsense	8	*RB1*_00063
122T	c.958C>T	p.Arg320*	Nonsense	10	*RB1*_00072
537T	c.958C>T	p.Arg320*	Nonsense	10	*RB1*_00072

LOVD- **L**eiden **O**pen (source) **V**ariation **D**atabase

#### Germline alterations (blood)

**Fig 5:**
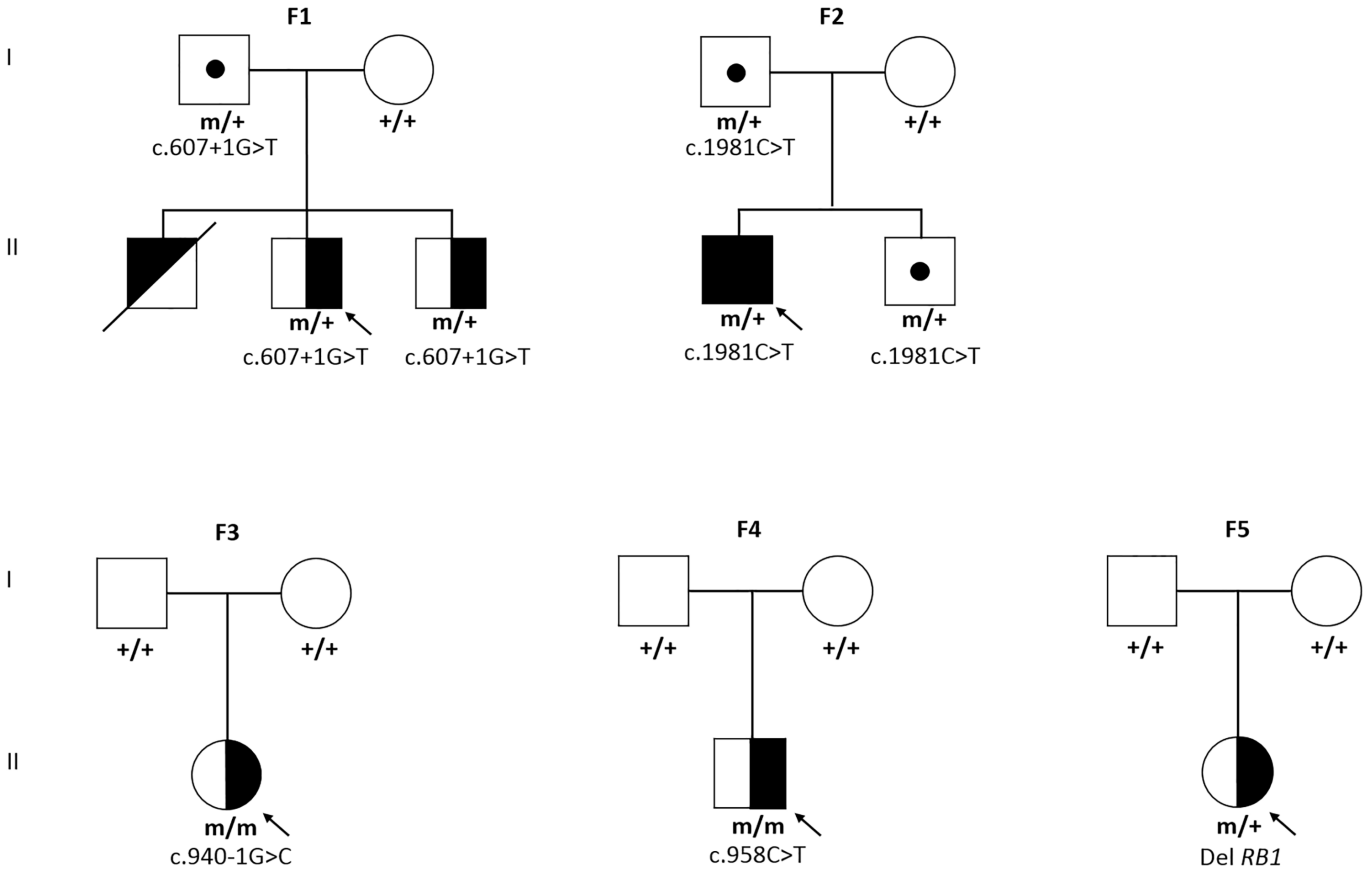
Pedigree of families with low penetrance and mosaic *RB1* mutations. Family **F1**: Father is a normal carrier of the heterozygous mutation: c.607+1G>T; Altered splicing. The proband and his younger brother carry the same heterozygous mutation and have unilateral RB. The proband also had an elder deceased sibling. Family **F2**: Father is an unaffected carrier of the heterozygous mutation: c.1981C>T; p.Arg661Trp. The proband and his brother have bilateral RB and carry the same heterozygous mutation. Family **F3**: The proband with unilateral RB carries a homozygous mutation: c.940-1G>C (altered splicing) in tumor. Only 4% of the proband’s blood leukocytes tested positive for the mutation (mosaicism). Both parents tested negative for the given mutation. Family **F4**: The proband with unilateral RB carries a homozygous mutation: c.958C>T; p.Arg320* in tumor. Only 2% of the proband’s blood leukocytes tested positive for the given mutation (mosaicism). Both parents tested negative for the given mutation. Family **F5**: The proband with unilateral RB has a deletion of one copy of *RB1* allele in tumor and a mosaicism for the same mutation in blood, as only 60% blood leukocytes carried the given mutation. Both parents tested negative for the given mutation. Genotype is provided for tested members as m/m for homozygous carriers, m/+ for heterozygous carriers and +/+ for homozygous wild-type. Blackened symbols: bilateral RB; half-blackened symbols: unilateral RB; diagonally blackened symbol: Unknown Laterality of RB; dotted symbols: unaffected carriers; dashed symbols: deceased.

Overall, 42.4% (25/59) cases tested positive for *RB1* mutations in the peripheral blood, which included 24 cases with point mutations and one case of gross deletion of germline origin (Table 5). All but one bilateral tumor presented with germline *RB1* mutations (17/18; 94.4%) (Fig 2). The remaining one bilateral case had a single somatic point mutation not present in blood, which suggests the presence of another germline *RB1* mutation which was not detected by the methods used in this study. Among the unilateral cases, 19.5% (8/41) were reported to harbour *RB1* mutations in blood. The frequencies of the different types of *RB1* germline point mutations (nonsense, frameshift, splicing, missense) are shown in Table 6. Further analysis of parental transmission of mutant alleles in all the 25 pairs of unaffected parents of cases with germline mutations revealed only two cases with positive transmission of the variant alleles (c.607+1G>T and c.1981C>T). Both mutations occurred in the two familial cases among our cohort. The origin of variant allele was paternal in both families, whereby one was a unilateral (family F1, Fig 5) case and the another, a bilateral (family F2, Fig 5) case. These mutations have previously been reported to cause low penetrance phenotype of RB and a summary of all reported cases harbouring similar (c.607+1G>T and c.1981C>T) low penetrance mutations is shown in Table 7. Within the bilateral cases, 5.6% (1/18) harboured a low penetrance phenotype.

**Table 5:** List of germline *RB1* mutations identified in blood.

Case	Laterality	Mutation	Putative Consequence	Mutation Type	Location	Reported (LOVD)
189T	Bilateral	**c.1568T>G (Homo)**	p.Leu523*	Nonsense	Exon 17	*RB1*_01352
208T	Bilateral	**c.1735_1736insGA (Homo)**	Gly581Lysfs*31	Frameshift insertion	Exon 18	Novel
280T	Bilateral	**c.958C>T (Homo)**	p.Arg320*	Nonsense	Exon 10	*RB1*_00072
308T	Bilateral	**c.225G>A (Homo)**	p.Trp75*	Nonsense	Exon 2	*RB1*_01495
367T	Bilateral	**c.175delG (Homo)**	p.Ala59Hisfs*5	Frameshift deletion	Exon 2	Novel
381T	Bilateral	**c.2494_2495delTT (Homo)**	p.Leu832Serfs*5 (C-terminus)	Frameshift deletion	Exon 24	Novel
432T	Bilateral	**c.224G>A (Homo)**	p.Trp75* (N-terminus)	Nonsense	Exon 2	*RB1*_00494
436T	Bilateral	**c.265-1G>T (Homo)**	Removal of acceptor site	Splicing	Intron 2	*RB1*_01476
572T	Bilateral	**c.1363C>T (Homo)**	p.Arg455*	Nonsense	Exon 14	*RB1*_00096
212T	Bilateral	c.2359C>T (Het)	p.Arg787*	Nonsense	Exon 23	*RB1*_00005
336T	Bilateral	c.265-2A>G (Het)	Removal of acceptor site	Splicing	Intron 2, In14	*RB1*_00322
423T	Bilateral	c.1363C>T (Het)	p.Arg455*	Nonsense	Exon 14	*RB1*_00096
462T	Bilateral	c.1981C>T (Het)	p.Arg661Trp	Missense	Exon 20	*RB1*_00019
545T	Bilateral	c.1494T>G (Het)	p.Tyr498*	Nonsense	Exon 16	*RB1*_00314
583T	Bilateral	c.1494T>G (Het)	p.Tyr498*	Nonsense	Exon 16	*RB1*_00314
592T	Bilateral	c.658C>G (Het)	Leu220Val	Missense	Exon 7	*RB1*_00251
604T	Bilateral	c.1510C>T (Het)	Gln504X	Nonsense	Exon 17	*RB1*_00668
111T	Unilateral	**c.2455C>G (Homo)**	p.Leu819Val	Missense	Exon 23	*RB1*_02045
227T	Unilateral	**c.763C>T (Homo)**	p.Arg255*	Nonsense	Exon 8	*RB1*_00063
519T	Unilateral	**c.940-1G>C (Homo)**	Altered splicing	Splicing (mosaic)	Intron 9	*RB1*_00195
578T	Unilateral	**c.958C>T (Homo)**	p.Arg320*	Nonsense (mosaic)	Exon 10	*RB1*_00072
182T	Unilateral	c.1654C>T (Het)	p.Arg552*	Nonsense	Exon 17	*RB1*_00121
477T	Unilateral	c.607+1G>T (Het)	Altered splicing	Splicing	Intron 6	*RB1*_00191
569T	Unilateral	c.1450_1451delAT (Het)	p.Met484Valfs*8;	Frameshift deletion	Exon 16	*RB1*_00105,
558T	Unilateral	-	-	Whole Gene deletion (mosaic)	-	

Total 24 *RB1* point mutations and 1 whole gene deletion were identified in 25 cases. Homozygous mutation is shown in bold.

**Table 6:** Spectrum of germline *RB1* point mutations detected in blood of probands.

Mutation Type	Unilateral	Bilateral	Total*
**Nonsense**	3	10	13/24 (54.17%)
**Frameshift**	1	3	4/24 (16.67%)
**Splicing**	2	2	4/24 (16.67%)
**Missense**	1	2	3/24 (12.5%)
**Distribution of germline mutation by laterality (%)**	7/24 (29.17%)	17/24 (70.83%)	24/24 (100%)

* A total of 24 germline point mutations were detected in blood from both unilateral and bilateral cases.

**Table 7:** Germline mutations identified in this study that were previously reported as low-penetrance mutations.

Mutation (Location)	Number of times reported as *RB1* mutation in LOVD	Cases previously reported as carriers of Low Penetrance mutation	Reference
c.1981C>T; p.Arg661Trp (Exon 20)	33	35	[34–37]
c.607+1G>T; Splicing (Intron 6)	21	19	[38–40]

LOVD- **L**eiden **O**pen (source) **V**ariation **D**atabase

Of the unilateral probands, who tested positive for *RB1* mutations in blood, 37.5% (3/8) were found to be mosaics. The three probands showed mosaicism for a splice site mutation - c.940-1G>C, 4% mosaic in blood (family F3, Fig 5); a nonsense mutation- p.Arg320*, 2% mosaic in blood (family F4, Fig 5) and a deletion of one *RB1* allele, 60% mosaic in blood (family F5, Fig 5), respectively. Parents of all 3 probands tested negative for *RB1* mutations (families F3-F5, Fig 5). A summary of the three mosaic mutations identified in this study, one of which has been previously described (c.958C>T), is given in Table 8.

**Table 8:** Germline mutations identified in this study that were reported as mosaic mutations.

Mutation (Location)	Number of times reported as mutations in LOVD	Cases identified as Mosaics in our study (% Mosaicism)#	Cases previously reported as mosaics (%Mosaicism)	Reference
c.958C>T; p.Arg320*; (Exon 10)	113	1 (2%)	3 (10%, <5%, 10%)	[41]
			1 (10%)	[42]
			1 (<50%)	[43]
c.940-1G>C (Intron 9)	2	1 (4%)	0	-
*RB1* whole gene deletion	NA	1 (60%)	0	

^#^ Levels of mosaicism reflected as % of mutant to wild-type allele as detected by allele-specific PCR in DNA from blood. LOVD- **L**eiden **O**pen (source) **V**ariation **D**atabase

#### Somatic alterations (tumor)

Among the different types of mutations arising only in the somatic cells, there were 37 which were point mutations (Table 9). These mutations were detected in 26 non-heritable cases which did not carry any germline mutation and in 6 heritable cases which carried one germline mutation in the tumor cell. The frequency of different types of mutations as observed within the somatic point mutations is shown in Table 10.

**Table 9:** List of somatic *RB1* point mutation identified only in tumor cells.

Case	Laterality	Mutation	Putative Consequence	Mutation Type	Location	Reported (LOVD)
143T	Unilateral	**c.1666C>T (Homo)**	p.Arg556*	Nonsense	Exon 17	*RB1_00124*
224T	Unilateral	**c.2174_2175insGT (Homo)**	-	Frameshift ins	Exon 21	Novel
232T	Unilateral	**c.1072C>T (Homo)**	p.Arg358*	Nonsense	Exon 11	*RB1_00008*
304T	Unilateral	**c.2359C>T (Homo)**	p.Arg787*	Nonsense	Exon 23	RB1_00005
320T	Unilateral	**c.1333C>T (Homo)**	p.Arg445*	Nonsense	Exon 14	RB1_00003
341T	Unilateral	**c.1399C>T (Homo)**	p.Arg467*	Nonsense	Exon 15	*RB1_00099*
345T	Unilateral	**c.1072C>T (Homo)**	p.Arg358*	Nonsense	Exon 11	*RB1_00008*
349T	Unilateral	**c.1959_1960insA (Homo)**	Val654Serfs*14	Frameshift ins	Exon 19	*RB1_01687*
378T	Unilateral	**c.1439_1441del (Homo)**	p.Asn480del	Frameshift del	Exon 16	*RB1_00102*
394T	Unilateral	**c.1333C>T (Homo)**	p.Arg445*	Nonsense	Exon 14	*RB1*_00003
410T	Unilateral	**c.-490A>T (Homo)**	-	Promoter	Upstream	Novel
414T	Unilateral	**c.2067G>C (Homo)**	p.Gln689His	Missense	Exon 20	Novel
420T	Unilateral	**c.1831A>T (Homo)**	p.Arg611*	Nonsense	Exon 19	Novel
435T	Unilateral	**c.1363C>T (Homo)**	p.Arg455*	Nonsense	Exon 14	*RB1_00096*
440T	Unilateral	**c.1654C>T (Homo)**	p.Arg552*	Nonsense	Exon 17	*RB1_00121*
550T	Unilateral	**c.1450_1451insAT (Homo)**	p.Met484Asnfs*12	Frameshift ins	Exon 16	*RB1_01736*
575T	Unilateral	**c.763C>T (Homo)**	p.Arg255*	Nonsense	Exon 8	*RB1*_00063
122T	Unilateral	c.1604_1605delTT (Het)	p.Phe535Tyrfs*1	Frameshift del	Exon 17	Novel
122T	Unilateral	c.958C>T (Het)	p.Arg320*	Nonsense	Exon 10	*RB1*_00072
150T	Unilateral	c.1363C>T (Het)	p.Arg455*	Nonsense	Exon 14	*RB1_00096*
182T#	Unilateral	c.301delA; (Het)	p.Ile101Serfs*9;	Frameshift del	Exon 3	*Novel*
210T	Unilateral	c.948_951delTCTT (Het)	p.Ser318Asnfs*13	Frameshift ins	Exon 10	*Novel*
244T	Unilateral	c.1333C>T (Het)	p.Arg445*	Nonsense	Exon 14	*RB1_00003*
244T	Unilateral	c.1072C>T (Het)	p.Arg358*	Nonsense	Exon 11	*RB1_00008*
329T	Unilateral	c.1494T>G (Het)	p.Tyr498*	Nonsense	Exon 16	*RB1*_00314
456T	Unilateral	c.1735delC (Het)	p.Arg579Glufs*32	Frameshift del	Exon 18	*RB1_00451*
456T	Unilateral	c.1653_1654insCG (Het)	p.Cys553Aspfs*59	Frameshift ins	Exon 17	*RB1_01738*
533T	Unilateral	c.1466G>A (Het)	p.Cys489Tyr	Missense	Exon 16	*RB1_00081*
533T	Unilateral	c.1150C>T (Het)	p.Gln384*	Nonsense	Exon 12	*RB1_01684*
537T	Unilateral	c.1735C>T (Het)	p.Arg579*	Nonsense	Exon 18	*RB1_00129*
537T	Unilateral	c.958C>T (Het)	p.Arg320*	Nonsense	Exon 10	*RB1_00072*
569T#	Unilateral	c.2106+2T>G (Het)	Altered splicing	Splicing	In 20	*RB1_01791*
212T#	Bilateral	c.1072C>T (Het)	p.Arg358*	Nonsense	Exon 11	*RB1_00008*
332T	Bilateral	c.958C>T (Het)	p.Arg320*	Nonsense	Exon 10	*RB1_00072*
336T#	Bilateral	c.1390-14A>G (Het)	Removal of acceptor site	Splicing		*RB1_00919*
545T#	Bilateral	c.1736_1745del10 (Het)	p.Arg579Glnfs*29	Frameshift del	Exon 18	*RB1_00014*
583T#	Bilateral	c.1736_1745del10 (Het)	p.Arg579Glnfs*29	Frameshift del	Exon 18	*RB1_00014*

^#^ Heritable cases with a germline point mutation detected in tumor cell. Homozygous mutation is shown in bold.

**Table 10:** Spectrum of somatic *RB1* point mutations detected only in retinoblastoma tumors in Singaporean cohort.

Mutation Type	Unilateral	Bilateral	Total*
Nonsense	19	2	21/37 (56.75%)
Frameshift	9	2	11/37 (29.73%)
Splicing	1	1	2/37 (5.4%)
Missense	2	0	2/37 (5.4%)
Promoter	1	0	1/37 (2.7%)
**Distribution of somatic mutation by laterality (%)**	32/37 (86.48%)	4/37 (10.8%)	**37/37 (100%)**

* A total of 37 somatic point mutations were detected in tumor only. Germline mutations found in both tumor and blood were excluded.

The remaining somatic alterations observed were *RB1* gross deletion and promoter methylation. There were 36 gross *RB1* deletions found in 47.45% (34/59) tumors, of which 9 were bilateral and 25 were unilateral. Of the cases with gross *RB1* deletions, only 26 cases could be further analysed for parental origin of the lost *RB1* allele due to limited availability of parental DNA. We found 61.5% (16/26) cases showing preferential loss of maternal allele, while 38.5% (10/16) cases showed preferential loss of paternal *RB1* allele (Table 11). The graphical representation of gross *RB1* deletions with respective origin of allelic loss is given in Fig 4. *RB1* promoter was found to be hypermethylated in only one unilateral case (410T), who also harboured a gross deletion within *RB1* gene (spanning Exons 17–24).

**Table 11:** Parental origin of allelic loss.

	Cases with Maternal allelic loss	Cases with Paternal allelic Loss	Total allelic loss
**Heritable**	7	3	10/26 (38.5%)
**Non-Heritable**	9	7	16/26 (61.5%)
**Total**	16/26 (61.5%)	10/26 (38.5%)	26/26 (100%)

Data from 26 cases in which parental samples were available for this analysis.

## Discussion

The spectrum of *RB1* mutations in cases diagnosed with RB in Singapore show small sequence mutations and gross *RB1* deletions as the major mechanisms of *RB1* inactivation. The majority of *RB1* point mutations are known to be distributed throughout the gene, with specific patterns of recurrent mutations and mutational hotspots encompassing retinoblastoma pocket domain coding regions: exons 12–23 [14,33,38,42,44–48]. We observed a high mutation rate in the sequence coding for these pocket domains within our cohort (58.1%) which was comparable to the previously reported rates of 58.6% [49] and 40% [42]. In addition, 44.3% (27/61) of the point mutations are known recurrent mutations and located in the CpG rich *RB1* mutational hotspots spanning exons 8, 10, 11, 14, 15, 17, 18 and 23. Point mutations in these 8 exons have been reported previously at variable frequencies of 50% (15/30) [50] and 35.7% (5/14) [51] in Chinese population. Of these identified recurrent mutations, seven belonged to a group of recurrent *RB1* CGA (Arg)>TGA(STOP) nonsense mutations (Arg255*, Arg320*, Arg358*, Arg445*, Arg455*, Arg552* and Arg787*) [41,52]. A meta-analysis on the *RB1* mutation spectrum across published databases previously revealed a 40% recurrent mutation frequency across 16 mutational hotspots in the CpG islands of *RB1* gene, out of which, 79% variants were associated with the recurrent C to T change in 11 CGA codons [33]. Within our cohort of 41 unilateral cases, 40 were sporadically affected and one was a familial case. We observed 17.5% (7/40) sporadic unilateral cases carrying germline *RB1* mutations which is comparable to previous reports indicating an incidence of between 10% - 18% [41,53–58]. The frequency of heritable and non-heritable *RB1* mutational events in this Singaporean cohort of 59 RB cases primarily comprised of familial or *de novo* sequence point mutations (50 cases), acquired intragenic and extragenic deletions (35 cases) and epigenetic changes (1 case). Although *RB1* promoter hypermethylation has been observed to play an important role as one of the two hits in RB with varying frequencies ranging from 0–27% [49,51,55,59,60]; we found only one instance of this, concurring with the previous observation by Choy et al 2002, that it is not a major inactivating mechanism in our population which had a predominance of Chinese patients. The unilateral patient with promoter hypermethylation in our study also harboured a novel somatic upstream *RB1* variant: c.-490A>T (Table 3 and S3 Table). This represents an incidence of 1.7% (1/59 cases) in the Singaporean population. Methylation of the *RB1* promoter is known to be the causative ‘first hit’ in about 8% of unilateral non-heritable tumours and about 88% of those were reported to have *RB1* gross deletions or loss of heterozygosity as ‘second hit’ [53]. The cumulative impact of the identified somatic variations occurring in the regulatory region of *RB1* in our proband is unknown as we were unable to perform any functional studies due to the samples from this case being depleted. However, based on previous *RB1* promoter methylation and sequence alteration studies [61,62], reduced gene expression of *RB1* can be postulated in such patients.

Occurrence of novel mutations can be as high as 20% to 53.3% of all mutations in patient populations of Chinese ethnicity reported from different countries [51,60,63,64]. In this study, we report 10 novel likely pathogenic variants which add to the genetic spectrum of RB disease. Reporting of novel variants is important as line of evidence for attributing pathogenicity when the same variants are subsequently found in other unrelated patients. This studied population with predominant Chinese ethnicity represents the first report from Singapore and contributes towards the reported variants in the literature.

### Tumors with one or no mutations identified

Among the 59 tumors, sixteen tumors presented with either one (13/59, 22.03%) or no *RB1* mutations (3/59, 5.1%) as shown in S4 Table. *RB1* independent means of tumorigenesis have been reported in a fraction of cases with somatic *MYCN* gene amplification [9,65]. These patients with unilateral *RB1*^+/+^*MYCN*^A^ retinoblastomas are usually diagnosed at a younger age (mean age = 4.5 months) with distinct histological features and harbour fewer genomic copy-number changes characteristic of retinoblastoma [9]. Aberrant methylation of *MGMT* has been suggested as an additional epigenetic dysregulation mechanism underlying retinoblastoma [66]. Tumor Necrosis Factor (*TNF*) gene is located on chromosome 6p, a region of recurrent chromosomal gain often observed in RB [14,65,67]. In RB, TNFs were found to be overexpressed in the membrane compartments and cytoplasm of WERI-Rb1 (with i(6p)) and Y79 (without i(6p)) retinoblastoma cell lines [67]. Of these 18 cases, we further analysed 10 samples which had sufficient remaining genomic DNA from tumor, for alterations in other candidate genes previously suspected to be associated with RB, viz. *MYCN* and *TNF* genes amplification and *MGMT* promoter methylation, using the screening methods described in this study. However, no *MYCN* amplification could be detected in any of the 10 analyzed RB cases. Hypermethylation of *MGMT* promoter was observed in 20% (2/10) cases, which included one case carrying both *TNF* gene amplification as well as *MGMT* promoter hypermethylation events (S5 Table). For *TNF* gene amplification analysis, 30% (3/10) retinoblastoma tumour samples harboured more than two copies of *TNF* gene (S5 Table).

In the remaining 5% (3/59) of cases, no *RB1* mutations were found. This could be due to the fact that conventional methods of *RB1* screening have limited scope as they might miss out on variants in deep intronic and untranslated regions (UTRs) of the gene, which could further be analyzed by incorporating other screening methods such as next generation sequencing technology [68]. A recent case of familial RB caused by retrotransposition of a Long interspersed element-1 (LINE-1) into the *RB1* gene also suggests that such events might be missed by most commonly used mutation detection platforms which are based on amplification of small fragments [69]. Thus, a combinatorial approach of RNA-based techniques and massive parallel sequencing is recommended for cases where no *RB1* alteration can be identified. Nevertheless, our mutation detection rate of 92.5% for unilateral tumors and 100% for bilateral tumors, are comparable to other studies that have employed direct sequencing and MLPA as a combinatorial approach towards determining the *RB1* mutational status with detection figures ranging between 92–100% for Bilateral and 10–61% in unilateral cases [45,50,55,70,71]. The high detection rates of *RB1* gene mutations reported in our study shows that conventional techniques are still effective as clinical screening methods for most RB cases even in the era of next-generation sequencing due to the type of mutations that occur frequently in this disease.

### Low-penetrance mutation

Low-penetrance phenotype in RB explains the phenomena of familial cases inheriting potentially deleterious mutated variants from an unaffected parent who lacks any disease related phenotype. Such asymptomatic carriers may have the proband’s mutation as a single recessive mutant allele or in some rare cases, even a dominant variant. However, they may present with a clinical phenotype which is either within the range of normal healthy variations, or is too mild to get noticed or would become apparent in later decades of life [72]. In addition, it was recently shown that both *RB1* gene and the upstream inhibitor of pRB: *CDKN1C* (cyclin-dependent kinase) gene are evolutionarily selected for maternal inhibition of cell proliferation [73]. Hence, this imprinting of *RB1* gene due to a differentially methylated CpG island in intron 2 results in parent-of-origin-specific DNA methylation and gene expression patterns [74]. We observed that both the identified familial cases in our cohort presented with low-penetrance retinoblastoma phenotype. The first case (Family F1; Fig 5) was a 2-year-old boy with unilateral RB who had inherited the low-penetrance splice site mutation (c.607+1G>T) from his asymptomatic father in a heterozygous state. Family history revealed that the proband had a deceased elder sibling (at 2-year-10-months of age) who was known to have metastasized RB tumor of unknown laterality and was not tested for the given mutation. The proband also had a younger brother (14-months) who presented with unilateral RB and harboured the same mutation. Typically, low-penetrance mutations are known to have a disease-eye ratio (*der*) ≤ 1, which is the ratio of the total number of affected eyes to the number of carriers in the family, while high penetrance mutations have *der* ≥1.5 ratio [75,76]. In family F1, *der* was found to be 0.66 (2/3) which concurs with the low-penetrance phenotype. To the best of our knowledge, our study reports the first Asian family (Family F1; Fig 5) harbouring c.607+1G>T low-penetrance mutation, which is known to present a variable expressivity phenotype in RB [38,39]. This mutation has been listed 21 times in the RB database (rb1-lsdb, Version *RB1* 150518) with a total of 19 individuals reported as carriers of this mutation [38–40]. Variable expressivity of this mutation is linked with the sex of the transmitting parent and was shown to cause RB in the progeny only when it is inherited from the father; as reported previously in Spanish [38] and German [39] patients. The second low-penetrance phenotype was due to the missense mutation: p.Arg661Trp, in family F2 (Family F2; Fig 5). The proband was diagnosed with bilateral RB at 10 months of age and was found to inherit the given germline mutation (heterozygous) from his normal father. His unaffected younger sibling’s DNA was analyzed and he was found to carry the same familial mutation in heterozygous state, thus leading to a *der* = 0.66 (2/3) in the family, which is according to the expected low-penetrance values. This low-penetrance mutation- p.Arg661Trp, has been reported 33 times in RB database (rb1-lsdb, Version *RB1* 150518) being present in 35 individuals [34–37] and its resultant protein is shown to have a temperature-sensitive pocket activity whose reversible fluctuations may result in low-penetrance phenotype [37]. A recent publication has linked this missense mutation to variable expressivity phenotype, with a parent-of-origin gender effect determining the probability of developing the disease [35]. The study reported that the probability of not developing RB was 90.3% when the mutation was inherited from the mother and 32.5% when inherited from father (p-value = 7.10^−7^) [35]. Thus, in families which carry the low-penetrance mutations, a reduced number of carriers develop retinoblastoma rather than the expected rate of >99% [43,52] which is commonly expected for most *RB1* mutations. Apart from the dominant inheritance pattern of RB, a few families have been reported to display a phenomena characterized by reduced penetrance and may not develop RB or may result in the development of unilateral RB or retinomas instead of bilateral RB (reduced expressivity) [76]. It has been hypothesised that reduction in the quantity or quality of cellular pRB activity is central to these low-penetrance mutations. In addition, pRB may be partially inactivated by subtle mutations that globally reduce the stability and binding affinity of the protein or that locally perturb semi essential functions [76]. Among the reasons postulated for this male-specific transmission of disease are differential regulation of genes in males and females [77] and *RB1* genomic imprinting [73]. In both our families carrying the reduced penetrance mutation, although the offsprings had retinoblastoma through paternal inheritance of the mutant alleles, there was reduced penetrance associated with the mutant alleles as fathers in the two families did not have the disease. Additionally, there also appears to be reduced expressivity in both siblings carrying the c.607+1G>T mutation as they presented with unilateral retinoblastoma and no further tumors were detected in follow-ups. Less than 10% of familial RB are known to show low penetrance phenotype. A meta-analysis from Valverde et al showed 20% (27/133) of all familial cases and 3.5% (27/753) of all germline *RB1* mutations to be linked to low penetrance phenotype in patients, whereby this distribution in familial cases was suggested to be an overestimation of the true incidence due to research bias for low penetrance phenotypes. In our smaller study, we encountered two such cases out of 25 cases bearing germline mutations suggesting that such mutations may not be that uncommon because they are recurrent mutations previously associated with low penetrant phenotype. For further identification of novel splice site and missense mutations that could be associated with low penetrance, genotype-phenotype correlations in larger families are necessary.

### Mosaic mutation

The phenomena of mosaicism in RB occurs when a mutation in the *RB1* gene arises at some time during embryogenesis -‘post zygotic’[43,78]. Therefore, a mosaic may have the initial mutation in some but not all cells of the body. Overall *RB1* mosaicism has been reported between 10–20% of all RB cases, previously [43]. Low-level germline mosaicism in sporadic bilateral RB has been reported in about 5% [41] cases, while a higher detection rate was achieved by using Deep Semiconductor Sequencing, 30% [58]. Around 12–14% of sporadic unilateral cases are known to carry *de novo*, germline mutations, of which 1.2% may be mosaics, and could be transmitted to the offspring [41,53]. The incidence of germline mosaicism in sporadic unilateral RB cases has been reported at varying frequencies of 3.8% [41], 6% [58], 8.7% [49], 16.6% [79], 22.2% [59] and 33% [42]. These varying frequencies observed are linked to the type of detection platform employed and the sample size of the study. A proband with sporadic RB possesses a 10% risk of bearing an undetectable low level *RB1* mosaicism when conventional genetic testing techniques fail to identify any *RB1* mutations [80]. In this study, we detected germline *RB1* mosaicism in three RB cases, which are all unilateral. The first patient (578T), who was diagnosed with unilateral RB at 26 months of age, carried 2% mosaicism for the stop-gain mutation in exon 10 (p.Arg320*). Mosaicism for this recurrent mutation- p.Arg320* has been reported previously in 5 cases (4 bilateral and 1 unilateral) at frequencies of <5, 10 and <50% (Table 8), whereby the unilateral case with unknown age carried a mosaicism at 10% frequency [41] (Table 8). The overall incidence of mosaicism among all tumors in our study was 5.1% (3/59), and 12% (3/25) among cases where mutation was found in peripheral blood. However, this may not reflect the true incidence of mosaicism in our patient population as mosaic mutations are not routinely screened in our set-up. While Sanger sequencing can detect some cases of mosaicism through recognition of unequally reduced heterozygous peaks on sequence traces, accurate detection of low level mosaicism requires sensitive technique such as allele specific amplification or next generation sequencing [41,58,78].

### Genetic counselling and disease management

The ultimate goal of retinoblastoma therapy is to ensure high survival rate while minimizing collateral damage to surrounding tissues and low recurrence of the disease through efficient genetic counselling. On one hand, owing to increased awareness and advancements in RB therapeutics, the overall survival rate in high-income countries has improved from <5% to 99%; significantly poor survival rates in low-income countries still remains a cause of concern [5,81]. Based on the genetic make-up of the individual, there are three major scenarios where an RB Patient would require lifelong follow-up for future family planning or to avoid any risk of secondary cancers: 1) germline *RB1* mutations, 2) germline *RB1* Mosaicism, or 3) low penetrance *RB1* Mutations. Identification of germline mosaicism is important as it could lead to an increased recurrence risk to future siblings. While the rate of second malignancies in retinoblastoma survivors with low-penetrance or mosaic *RB1* mutations is still unknown, it is presumed to be lower than those with germline null *RB1* alleles [8]. A recent study reported cumulative incidence of developing a second malignancy by the age of 10 in patients with heterozygous germline *RB1* alterations was 5.2% (95%CI 1.7; 8.7%) [82]. One of the existing challenges of genetic counselling in retinoblastoma is the applicability of the identified heterogeneous spectrum of mutations in the patient which may in turn lead to variable disease phenotypes viz., low penetrance and mosaicism. Thus, efficient genetic testing would add to the growing knowledge and enable accurate genetic counselling with customized follow-up schedules for cases where the underlying mutation heterogeneity could be easily missed either due to a low-penetrance phenotype or because only a fraction of cells harbour the causative mutations (mosaicism). Hence, it is imperative to provide life-long follow-up to both the patients and their families with heritable mutations as the former carry a higher risk for developing secondary cancers and a probability of passing risk mutations to their future offspring, while the latter may require advice on family planning due to their asymptomatic carrier status.

With respect to therapeutics, disease outcomes have improved due to intra-arterial and intravitreal chemotherapy which are focused on salvaging the eyes, which otherwise would have been lost in conventional treatment [5]. Since the first successful report of preimplantation genetic testing [83], this preventive intervention appears promising for future disease management in families at risk for having children with inherited retinoblastoma. With respect to *RB1* mutational signature, since nonsense mutations comprise majority of the reported point mutations in RB, as also shown in our study, nonsense suppression therapy which allows readthrough of premature termination codon (PTCs), restoring the protein function [84]; offers possible future targeted therapeutics of such cases. Generation 4 polyamidoamine (G4PAMAM) dendrimers, which act as delivery system of vascular endothelial growth factor antisense oligodeoxynucleotides were recently reported to have antitumor properties, both *in vitro* and *in vivo* [85]. In addition to gene therapy, few sustained drug release platforms are also being developed for targeted intraocular drug delivery in RB [6]. Thus, information of the mutational signatures in RB patients would further aid in targeted therapeutics besides ensuring effective disease management and life-long follow up, where indicated.

## Conclusions

Our report expands the spectrum of *RB1* mutations and further emphasizes on the need to not only identify the causative mutations but also to detect special disease phenotypes viz., low-penetrance mutations and germline mosaicism. Thus, our study on identifying the genetic signatures from Singaporean patients with RB will further aid in developing appropriate screening programmes and devising efficient disease management measures for such patients and their families.

## Supporting information

S1 Table Sequences of primers for extragenic microsatellite markers used in this study. (DOCX)

S2 Table Sequences of primers for intragenic *RB1* markers used in this study. (DOCX)

S3 Table Overview of *RB1* mutations identified in total 59 Retinoblastoma cases. (DOCX)

S4 Table List of RB cases with number of *RB1* mutations. (DOCX)

S5 Table List of 10 cases analyzed for *TNF* and *MGMT* mutations, where only one or no *RB1* mutations could be identified. (DOCX)
