# Supplementary material for: Mutation spectrum of RB1 mutations in retinoblastoma cases from Singapore with implications for genetic management and counselling
Source: PLoS One. 2017 Jun 2;12(6):e0178776. doi: 10.1371/journal.pone.0178776 (PMC5456385; doi:10.1371/journal.pone.0178776)
Supplement: S1 Table — (DOCX) [file pone.0178776.s001.docx]

**S1 Table. Sequences of primers for extragenic microsatellite markers used in this study**

| Marker | Genomic location | Primer Sequence | | Amplicon Size (bp) | Tm (°C) |
| --- | --- | --- | --- | --- | --- |
| D13S1316 | 13pter-13qter | CTACTGGGGAGGCTGG | GGCCTGAAAGGTATCCTC | 257-265 | 52 |
| D13S1236 | 13pter-13qter | GCACTTGGCCTGGGTAA | AAGGGGCTGGCTCTTCA | 124-132 | 55 |
| D13S175 | 13q11-13q11 | TATTGGATACTTGAATCTGCTG | TGCATCACCTCACATAGGTTA | 101-113 | 55 |
| D13S221 | 13q12.1-13q12.1 | TAGCCATGATAGGAAATCAACC | GAGATCGTGCAGCACTTGT | 223-243 | 55 |
| D13S260 | 13q12.3-13q12.3 | AGATATTGTCTCCGTTCCATGA | CCCAGATATAAGGACCTGGCTA | 158-173 | 52 |
| D13S171 | 13q12.3-13q13 | CCTACCATTGACACTCTCAG | TAGGGCCATCCATTCT | 227-241 | 55 |
| D13S267 | 13q12.3-13q12.3 | GGCCTGAAAGGTATCCTC | TCCCACCATAAGCACAAG | 148-162 | 55 |
| D13S220 | 13q12.3-13q13 | CCAACATCGGGAACTG (6-FAM) | TGCATTCTTTAAGTCCATGTC | 191-203 | 55 |
| D13S218 | 13q13-13q14.1 | GATTTGAAAATGAGCAGTCC | GTCGGGCACTACGTTTATCT | 187-195 | 55 |
| D13S328 | 13q14.2-13q14.3 | TGTAGCCCTTGACTAGAATCAC | GATAGGTCAGTTGAAATCTCTACG | 244-258 | 55 |
| D13S263 | 13q14.1-13q14.2 | CCTGGCCTGTTAGTTTTTATTGTTA | CCCAGTCTTGGGTATGTTTTTA | 149 | 55 |
| D13S284 | 13q14.3-13q14.3 | AAAATCAGGTGGAAACAGAAT | AAAGGCTAACATCGAAGGGA | 208-230 | 55 |
| D13S319 | 13q14.3-13q14.3 | CGAGCTGGAGTCCATCGTAT | CGTCGCTGCAGATCAAAGGA | 168-182 | 52 |
| D13S137 | 13q14.3-13q14.3 | CAGGAGGGATGGACTCACTTC | TTTCCTCATTCTTTCCCCAATTG | 113-135 | 53 |
| D13S170 | 13q31-13q31 | TTGCACTGTGGAGATAAACACATAG | TCACATTGTCTTTTAAGGCAGGAG | 113-137 | 52 |
| D13S282 | 13q31-13q32 | ATAGCCTGTGAAGTCTCTGCAT | TCAACTATCTTTGGACTGAAAGTG | 236 | 55 |
| D13S154 | 13q31-13q32 | GTGCTATAAAGGCTTGCTGC | CTCTTGCCCTGGTCTTGACT | 256 | 55 |
| D13S280 | 13q32-13q32 | CGG AATTACTGCCTGGGGTGAG | AGGAGCGTATGACATC | 241 | 52 |
| D13S285 | 13q34-13q34 | ATATATGCACATCCATCCATG | GGCCAAAGATAGATAGCAAGGTA | 100 | 52 |
| D13S293 | 13q34-13q34 | GCTGTTTGGATCTCCC | TCCTGTGAAACTTGTTTTG | 94 | 55 |
