# Supplementary material for: Mutation spectrum of RB1 mutations in retinoblastoma cases from Singapore with implications for genetic management and counselling
Source: PLoS One. 2017 Jun 2;12(6):e0178776. doi: 10.1371/journal.pone.0178776 (PMC5456385; doi:10.1371/journal.pone.0178776)
Supplement: S2 Table — (DOCX) [file pone.0178776.s002.docx]

**S2 Table. Sequences of primers for intragenic *RB1* markers used in this study**

| **Marker** | **Genomic location** | ***RB1* Location** | **Forward Primer** | **Reverse Primer** | **Amplicon Size (bp)** | **Tm°C** |
| --- | --- | --- | --- | --- | --- | --- |
| D13S153 | chr13:48890856-48890920 | Intron 2 | AGCATTGTTTCATGTTGGTG | CAGCAGTGAAGGTCTAAGCC | 212-236 | 58 |
| 22xTG | chr13:48920507-48920550 | Intron 4 | CGACCAAAATGTCAACAGTGG | GCTACCAGATATGAATTAAAAAGG | 221-227 | 56 |
| 16xTTCT | chr13:49034023-49034086 | Intron 20 | GTATGAACTCATGAGAGACAGGCAT | AATTAACAAGGTGTGGTGGTACACG | 344-360 | 63 |
| rs2854351 | chr13:48878271 | Intron 1 | CAGGACAGCGGCCCGGAG | CTGCAGACGCTCCGCCGT | 180 | 62 |
| rs368025410 | chr13:48976514 | Intron 17 | TTCCAATGAAGAACAAATGG | GCAATTGCACAACCAAGTT | 975 | 56 |
| rs4151620 | chr13:49049059 | Intron 24 | ACCTTCCTAGCACTTAGACA | AAGGGCAAGAAACCAATAGA | 1130 | 58 |
| rs3092904 | chr13:49051481 | Intron 25 | TCCATTTATAAATACACATG | TAACGAAAAGACTTCTTGCA | 150 | 53 |

The chromosomal locations of the markers are given as per GrCh37 (Hg19) build
