# Supplementary material for: Mutation spectrum of RB1 mutations in retinoblastoma cases from Singapore with implications for genetic management and counselling
Source: PLoS One. 2017 Jun 2;12(6):e0178776. doi: 10.1371/journal.pone.0178776 (PMC5456385; doi:10.1371/journal.pone.0178776)
Supplement: S3 Table — (DOCX) [file pone.0178776.s003.docx]

**S3 Table: Overview of *RB1* mutations identified in total 59 Retinoblastoma cases**

| Case | Gender | Age at diagnosis (months) | Laterality | Change in cDNA sequence | Putative Consequence | Mutation Type | Site | Present in Blood | Reported previously in *RB1* LOVD |
| --- | --- | --- | --- | --- | --- | --- | --- | --- | --- |
| 378T | M | 19 | Unilateral | c.1439_1441del (Homo) | p.Asn480del | Frameshift deletion | Exon 16 | No | RB1_00102 |
| 111T | F | 22 | Unilateral | c.2455C>G (Homo) | p.Leu819Val | Missense | Exon 23 | Yes | RB1_02045 |
|  |  |  |  | - | - | Gross deletion | - | No | - |
| 143T | M | 36 | Unilateral | c.1666C>T (Homo) | p.Arg556* | Nonsense | Exon 17 | No | RB1_00124 |
|  |  |  |  | - | - | Gross deletion | - | No | - |
| 224T | F | 18 | Unilateral | c.2174_2175insGT (Homo) | - | Frameshift inertion | Exon 21 | No | Novel |
|  |  |  |  | - | - | Whole Gene deletion | - | No | - |
| 227T | F | 0.27 | Unilateral | c.763C>T (Homo) | p.Arg255* | Nonsense | Exon 8 | Yes | RB1_00063 |
|  |  |  |  | - | - | Gross deletion | - | No | - |
| 232T | F | 15 | Unilateral | c.1072C>T (Homo) | p.Arg358* | Nonsense | Exon 11 | No | RB1_00008 |
|  |  |  |  | - | - | Gross deletion | - | No | - |
| 304T | M | 38 | Unilateral | c.2359C>T (Homo) | p.Arg787* | Nonsense | Exon 23 | No | RB1_00005 |
|  |  |  |  | - | - | Gross deletion | - | No | - |
| 320T | M | 28 | Unilateral | c.1333C>T (Homo) | p.Arg445* | Nonsense | Exon 14 | No | RB1_00003 |
|  |  |  |  | - | - | Whole Gene deletion | - | No | - |
| 341T | F | 72 | Unilateral | c.1399C>T (Homo) | p.Arg467* | Nonsense | Exon 15 | No | RB1_00099 |
|  |  |  |  | - | - | Whole Gene deletion | - | No | - |
| 345T | F | 5 | Unilateral | c.1072C>T (Homo) | p.Arg358* | Nonsense | Exon 11 | No | RB1_00008 |
|  |  |  |  | - | - | Whole Gene deletion | - | No | - |
| 349T | M | 10 | Unilateral | c.1959_1960insA (Homo) | p.Val654Serfs*14 | Frameshift inertion | Exon 19 | No | RB1_01687 |
|  |  |  |  |  |  | Whole Gene deletion | - | No | - |
| 394T | F | 42 | Unilateral | c.1333C>T (Homo) | p.Arg445* | Nonsense | Exon 14 | No | RB1_00003 |
|  |  |  |  | - | - | Gross deletion | - | No | - |
| 410T | M | 35 | Unilateral | c.-490A>T (Homo) | - | Promoter | Upstream | No | Novel |
|  |  |  |  | - | - | Gross deletion | - | No | - |
| 414T | F | 2 | Unilateral | c.2067G>C (Homo) | p.Gln689His | Missense | Exon 20 | No | Novel |
|  |  |  |  | - | - | Gross deletion | - | No | - |
| 420T | F | 17 | Unilateral | c.1831A>T (Homo) | p.Arg611* | Nonsense | Exon 19 | No | Novel |
|  |  |  |  | - | - | Gross deletion | - | No | - |
| 435T | F | 37 | Unilateral | c.1363C>T (Homo) | p.Arg455* | Nonsense | Exon 14 | No | RB1_00096 |
|  |  |  |  | - | - | Gross deletion | - | No | - |
| 440T | M | 36 | Unilateral | c.1654C>T (Homo) | p.Arg552* | Nonsense | Exon 17 | No | RB1_00121 |
|  |  |  |  | - | - | Gross deletion | - | No | - |
| 519T^#^ | F | 1 | Unilateral | c.940-1G>C (Homo) | Altered splicing | Splicing (mosaic) | Intron 9 | Yes | RB1_00195 |
|  |  |  |  | - | - | Gross deletion | - | No | - |
| 550T^#^ | F | 14 | Unilateral | c.1450_1451insAT (Homo) | p.Met484Asnfs*12 | Frameshift inertion | Exon 16 | No | RB1_01736 |
|  |  |  |  | - | - | Whole Gene deletion | - | No | - |
| 575T^#^ | F | 22 | Unilateral | c.763C>T (Homo) | p.Arg255* | Nonsense | Exon 8 | No | RB1_00063 |
|  |  |  |  | - | - | Gross deletion | - | No | - |
| 578T^#^ | M | 26 | Unilateral | c.958C>T (Homo) | p.Arg320* | Nonsense (mosaic) | Exon 10 | Yes | RB1_00072 |
|  |  |  |  | - | - | Gross deletion | - | No | - |
| 122T | F | NA | Unilateral | c.1604_1605delTT (Het) | p.Phe535Tyrfs*1 | Frameshift deletion | Exon 17 | No | Novel |
|  |  |  |  | c.958C>T (Het) | p.Arg320* | Nonsense | Exon 10 | No | RB1_00072 |
| 182T | F | NA | Unilateral | c.1654C>T (Het) | p.Arg552* | Nonsense | Exon 17 | Yes | RB1_00121 |
|  |  |  |  | c.301delA; (Het) | p.Ile101Serfs*9; | Frameshift deletion | Exon 3 | No | Novel |
| 244T | F | 48 | Unilateral | c.1333C>T (Het) | p.Arg445* | Nonsense | Exon 14 | No | RB1_00003 |
|  |  |  |  | c.1072C>T (Het) | p.Arg358* | Nonsense | Exon 11 | No | RB1_00008 |
| 456T^#^ | M | 9 | Unilateral | c.1653_1654insCG (Het) | p.Cys553Aspfs*59 | Frameshift inertion | Exon 17 | No | RB1_01738 |
|  |  |  |  | c.1735delC (Het) | p.Arg579Glufs*32 | Frameshift deletion | Exon 18 | No | RB1_00451 |
| 533T^#^ | M | 36 | Unilateral | c.1150C>T (Het) | p.Gln384* | Nonsense | Exon 12 | No | RB1_01684 |
|  |  |  |  | c.1466G>A (Het) | p.Cys489Tyr | Missense | Exon 16 | No | RB1_00081 |
| 537T^#^ | F | 36 | Unilateral | c.1735C>T (Het) | p.Arg579* | Nonsense | Exon 18 | No | RB1_00129 |
|  |  |  |  | c.958C>T (Het) | p.Arg320* | Nonsense | Exon 10 | No | RB1_00072 |
| 569T^#^ | F | 5 | Unilateral | c.1450_1451delAT (Het) | p.Met484Valfs*8; | Frameshift deletion | Exon 16 | Yes | RB1_00105 |
|  |  |  |  | c.2106+2T>G (Het) | Altered splicing | Splicing | Intron 20 | No | RB1_01791 |
| 150T | M | 23 | Unilateral | c.1363C>T (Het) | p.Arg455* | Nonsense | Exon 14 | No | RB1_00096 |
| 210T | F | 72 | Unilateral | c.948_951delTCTT (Het) | p.Ser318Asnfs*13 | Frameshift inertion | Exon 10 | No | Novel |
| 329T | M | 34 | Unilateral | c.1494T>G (Het) | p.Tyr498* | Nonsense | Exon 16 | No | RB1_00314 |
| 477T | M | 24 | Unilateral | c.607+1G>T (Het) | Altered splicing | Splicing (Low Pen) | Intron 6 | Yes | RB1_00191 |
| 326T | M | 42 | Unilateral | - | - | Whole gene deletion | - | No | - |
|  |  |  |  | - | - | Whole Gene deletion | - | No | - |
| 450T^#^ | M | 7 | Unilateral | - | - | Whole Gene deletion | - | No | - |
|  |  |  |  | - | - | Whole Gene deletion | - | No | - |
| 323T | M | 29 | Unilateral | - | - | Gross deletion | - | No | - |
| 352T | M | 22 | Unilateral | - | - | Gross deletion | - | No | - |
| 453T | F | 24 | Unilateral | - | - | Gross Del | - | No | - |
| 558T^#^ | F | 12 | Unilateral | - | - | Whole Gene deletion (mosaic) | - | Yes | - |
| 277T | M | 8 | Unilateral | - | - | - | - | No | - |
| 417T | F | 2 | Unilateral | - | - | - | - | No | - |
| 523T^#^ | F | 53 | Unilateral | - | - | - | - | No |  |
| 189T | F | NA | Bilateral | c.1568T>G (Homo) | p.Leu523* | Nonsense | Exon 17 | Yes | RB1_01352 |
|  |  |  |  | - | - | Whole Gene deletion |  | No |  |
| 208T | M | 6 | Bilateral | c.1735_1736insGA (Homo) | p.Gly581Lysfs*31 | Frameshift inertion | Exon 18 | Yes | Novel |
|  |  |  |  | - | - | Gross deletion |  | No |  |
| 280T | M | 11 | Bilateral | c.958C>T (Homo) | p.Arg320* | Nonsense | Exon 10 | Yes | RB1_00072 |
|  |  |  |  | - | - | Whole Gene deletion |  | No |  |
| 308T | F | 8 | Bilateral | c.225G>A (Homo) | p.Trp75* | Nonsense | Exon 2 | Yes | RB1_01495 |
|  |  |  |  | - | - | Whole Gene deletion |  | No |  |
| 367T | F | 29 | Bilateral | c.175delG (Homo) | p.Ala59Hisfs*5 | Frameshift deletion | Exon 2 | Yes | Novel |
|  |  |  |  | - | - | Gross deletion |  | No |  |
| 381T | F | 21 | Bilateral | c.2494_2495delTT (Homo) | p.Leu832Serfs*5 (C-terminus) | Frameshift deletion | Exon 24 | Yes | Novel |
|  |  |  |  | - | - | Gross deletion |  | No |  |
| 432T | M | 22 | Bilateral | c.224G>A (Homo) | p.Trp75* (N-terminus) | Nonsense | Exon 2 | Yes | RB1_00494 |
|  |  |  |  | - | - | Gross deletion |  | No |  |
| 436T | F | 13 | Bilateral | c.265-1G>T (Homo) | Removal of acceptor site | Splicing | Intron 2 | Yes | RB1_01476 |
|  |  |  |  | - | - | Gross deletion |  | No |  |
| 572T | F | 21 | Bilateral | c.1363C>T (Homo) | p.Arg455* | Nonsense | Exon 14 | Yes | RB1_00096 |
|  |  |  |  | - | - | Gross deletion |  | No |  |
| 212T | M | 23 | Bilateral | c.1072C>T (Het) | p.Arg358* | Nonsense | Exon 11 | No | RB1_00008 |
|  |  |  |  | c.2359C>T (Het) | p.Arg787* | Nonsense | Exon 23 | Yes | RB1_00005 |
| 336T | M | 3 | Bilateral | c.1390-14A>G (Het) | Removal of acceptor site | Splicing | Intron 14 | No | RB1_00919 |
|  |  |  |  | c.265-2A>G (Het) | Removal of acceptor site | Splicing | Intron 2 | Yes | RB1_00322 |
| 545T^#^ | F | 12 | Bilateral | c.1494T>G (Het) | p.Tyr498* | Nonsense | Exon 16 | Yes | RB1_00314 |
|  |  |  |  | c.1736_1745del10 (Het) | p.Arg579Glnfs*29 | Frameshift deletion | Exon 18 | No | RB1_00014 |
| 583T | F | 46 | Bilateral | c.1494T>G (Het) | p.Tyr498* | Nonsense | Exon 16 | Yes | RB1_00314 |
|  |  |  |  | c.1736_1745del10 (Het) | p.Arg579Glnfs*29 | Frameshift deletion | Exon 18 | No | RB1_00014 |
| 332T | M | 3 | Bilateral | c.958C>T (Het) | p.Arg320* | Nonsense | Exon 10 | No | RB1_00072 |
| 423T | F | 3 | Bilateral | c.1363C>T (Het) | p.Arg455* | Nonsense | Exon 14 | Yes | RB1_00096 |
| 462T^#^ | M | 10 | Bilateral | c.1981C>T (Het) | p.Arg661Trp | Missense (Low Pen) | Exon 20 | Yes | RB1_00019 |
| 592T | F | 24 | Bilateral | c.658C>G (Het) | p.Leu220Val | Missense | Exon 7 | Yes | RB1_00251 |
| 604T^#^ | F | 5 | Bilateral | c.1510C>T (Het) | p.Gln504* | Nonsense | Exon 17 | Yes | RB1_00668 |

F-Female, M-Male, NA- Not available

Low Pen-Variant known to have low penetrance phenotype in RB.

*RB1* mutations are listed according to the HGVS format and genomic nucleotide sequence L11910.1. The pRb domain affected is derived from NP_000312.2 and the cDNA change is derived from NM_000321.2. Previously identified *RB1* mutations are given as per the *RB1* Leiden Open (source) Variation Database (LOVD) version RB1 150518 (rb1-lovd.d-lohmann.de).

**^#^***Cases analysed by RB Solutions/Impact Genetics.*
