# Supplementary material for: Mutation spectrum of RB1 mutations in retinoblastoma cases from Singapore with implications for genetic management and counselling
Source: PLoS One. 2017 Jun 2;12(6):e0178776. doi: 10.1371/journal.pone.0178776 (PMC5456385; doi:10.1371/journal.pone.0178776)
Supplement: S4 Table — (DOCX) [file pone.0178776.s004.docx]

**S4 Table. List of RB cases with number of *RB1* mutations**

| No. of *RB1* Mutations | Category of Tumor | Unilateral probands (N = 41) | Bilateral probands (N = 18) | Total probands (N = 59) | (%) |
| --- | --- | --- | --- | --- | --- |
| **2 Hits** | with two point mutations | 9 | 4 | 13 | 22 |
|  | with one point mutation and one gross deletion | 18 | 9 | 27 | 45.8 |
|  | with two gross deletions | 2 | 0 | 2 | 3.4 |
|  | with one gross deletion and one promoter methylation | 1 | 0 | 1 | 1.7 |
|  | **Total cases with biallelic hits (N = 59)** | **30** | **13** | **43** | **72.9** |
| **1 Hit** | with one gross deletions only | 4 | 0 | 4 | 6.8 |
|  | with one point mutation only | 4 | 5 | 9 | 15.3 |
|  | **Total cases with single allelic hit (N = 59)** | **8** | **5** | **13** | **22.0** |
| **0 Hit** | **with no mutations found** | **3** | **0** | **3** | **5.1** |
