# Supplementary material for: Mutation spectrum of RB1 mutations in retinoblastoma cases from Singapore with implications for genetic management and counselling
Source: PLoS One. 2017 Jun 2;12(6):e0178776. doi: 10.1371/journal.pone.0178776 (PMC5456385; doi:10.1371/journal.pone.0178776)
Supplement: S5 Table — (DOCX) [file pone.0178776.s005.docx]

**S5 Table. List of 10 cases analyzed for *TNF* and *MGMT* mutations, where only one or no *RB1* mutations could be identified.**

| **Case** | **Laterality** | ***TNF* amplification** | ***MGMT* promoter hyper-methylation** | ***RB1* Gross Deletion** | ***RB1* Point Mutation** |
| --- | --- | --- | --- | --- | --- |
| 277T | U | - | - | - | - |
| 417T | U | - | - | - | - |
| 150T | U | - | - | - | + |
| 210T | U | + | - | - | + |
| 329T | U | + | + | - | + |
| 332T | B | - | + | - | + |
| 378T | U | - | - | - | + |
| 423T | B | + | - | - | + |
| 323T | U | - | - | + | - |
| 326T | U | - | - | + | - |

“+” indicates presence and “-” indicates absence of mutation/ gene amplification/ promoter hypermethylation.
